# Morbid Appearances after Death, (Exclusive of Intestinal Lesions,) Distinctive of the Typhoid and Typhus Types of Continued Fever

**Published:** 1852-08

**Authors:** 


ART. III. — *Morbid Appearances after Death, (exclusive of intestinal lesions,) distinctive of the Typhoid and Typhus types of Continued Fever.* By THE EDITOR.

The investigations upon which were based my Clinical Reports on Continued Fever relate chiefly to events belonging to the *living* history of the disease, that is to say, to the symptoms and circumstances developed during the continuance of life. In the fatal cases, inquiries concerning the morbid effects impressed upon the organism, were generally prosecuted but to a limited extent, attention frequently being directed only to the presence or absence of the intestinal lesions regarded as characteristic of the Typhoid type. It is needless to say that the appearances after death form an important portion of the natural history of any disease; and with reference to these, as well as the phenomena manifested during life, it is important to examine *all* the organs of the body, noting the absence, as well as presence of morbid changes. The post mortem history, like that pertaining to the phenomena of life, is to be based on analyses of recorded observations, sufficient in number, and made at different periods and places. Studied in this way, the morbid anatomy of Continued Fever offers a field for inquiry, as yet by no means exhausted. The researches of Louis embrace an account of the appearances in a pretty large number of cases of *Typhoid* fever; and within the past few years, facts have been contributed by various observers relative to the changes distinguishing this from the *Typhus* type. A point of absorbing interest, however, has been the presence of intestinal lesions in *Typhoid,* and their absence, or relative insignificance in *Typhus.* Attention appears to have been in a great degree engrossed by these lesions, to the exclusion of a more extended comparison of the two types embracing the appearances found in other organs.

Dr. William Jenner, Prof. of pathological anatomy in University College, London, has lately contributed some observations on this subject which are highly interesting and valuable. They are contained in a series of articles communicated for the *Monthly Journal of Medical Science*, thus entitled:— "On Typhoid and Typhus fevers — an attempt to determine the question of their identity or non-identity, by an analysis of the symptoms, and of the appearances found after death in sixty-six fatal cases of Continued Fever, observed at the London fever hospital from Jan., 1847, to Feb., 1849." It will be perceived by the foregoing title that the articles embrace an analysis of the symptoms, as well as post mortem appearances. The course pursued by Dr. J. was to select only fatal cases, and to determine the type by the presence or absence of the intestinal typhoid lesions. Having in this way arranged them into two groups, he then proceeded to analyze the symptoms and appearances after death in both groups, and compare the results. The articles by Dr. Jenner have fallen under my notice since my third Report was written. The data for comparison of the morbid appearances in the two types (typhus and typhoid) are fuller and more complete than, so far as I know, have been contributed by any other observer; and in order to supply a manifest deficiency in the analyses which I have made, I shall take the liberty of appropriating, for the benefit of those who have honored my Reports with a perusal, a summary of the results of his investigations. The synopsis which follows is copied from the Journal already mentioned, and is given as it is there presented by the author. The intestinal lesions are excluded. The propriety of this is apparent when it is considered that upon the presence or absence of these lesions the post mortem diagnosis was based. Moreover, as respects these lesions, the fatal cases in my collections were studied with considerable care.

*Synopsis of morbid appearances distinctive of Typhus and Typhoid fever, based on an analysis of sixty-six cases.*

" *Cadaveric rigidity,* ceased much more quickly in subjects dead from typhus than from typhoid fever."

" *Discoloration of the walls of the abdomen and of the skin covering the larger veins,* was much more frequently present in those dead from typhus than typhoid fever."

*Emaciation* had made greater progress in the typhoid than in the typhus subjects."

" *Spots.* The spots observed during the progress of the cases of typhus fever continued after death; no trace of the spots visible during life could be detected after death from typhoid fever."

" *Head.* After typhoid fever, the pia mater and arachnoid separated from the convolutions with abnormal facility in one only of nine cases examined with reference to this point. The vessels of the pia mater were abnormally filled with blood in one-third of the cases, but intensely congested in one only of the fifteen cases; the cerebral substance was congested in one-seventh of the cases. After typhus fever, the pia mater and arachnoid separated with abnormal facility in nine of eleven cases of which notes on the point were made. The vessels of the pia mater were congested in nearly half, and intensely congested in one-fifth of the whole of the cases; while the cerebral substance itself was abnormally congested in half."

"*Hemorrhage into the cavity of the arachnoid*, which was not found in a single case of typhoid fever, had occurred before death in one-eighth of the cases of typhus fever." "The amount of sesosity found within the cranial cavity was decidedly greater after typhus than typhoid fever."

"*Pharynx.* After typhoid fever, this organ was found ulcerated in one-third of the cases. After typhus fever, ulceration of the pharynx was not detected in a single case."

"*Larynx.* Ulceration of the larynx was found in one of fifteen subjects dead from typhoid fever; in one of twenty-six from typhus fever."

"*Œsophagus.* After typhoid fever, ulcerated in one of fifteen cases in which it was examined. After typhus fever, the œsophagus was free from ulceration in all the twenty-four cases in which it was examined."

"The epithelium separated from the œsophagus spontaneously at an earlier period after death from the latter, than the former disease."

"*Stomach.* In none of the fifteen cases examined after death from typhoid fever was the mucous membrane of the stomach softened throughout the whole extent; in no case did softening of the cardial extremity approach perforation. In four of thirty-seven cases of typhus fever the whole mucous membrane of the stomach was softened; and in four others there was such extreme softening of the whole of the coats of the great *cul de sac*, that they were perforated by the slightest violence.

"*Small Intestines and Mesenteric Glands.* The presence or absence of lesion of these organs was the ground on which the cases of typhoid and typhus fever here analyzed were divided from each other,—consequently they were invariably diseased in the one, and sound in the other."

"*Large Intestines.* After death from typhoid fever, the mucous membrane of the large intestines was found ulcerated in rather more than a third of twenty cases. In no instance after death from typhus fever.

"*Peritoneum.* As peritonitis was in typhoid fever secondary to, and dependent on the entero-mesenteric disease, it may here be excluded from consideration."

"*Spleen.* This organ was enlarged in all the cases of typhoid fever—softened in one-third of the cases only. Before the age of 50, it was as large after typhus as typhoid fever; after that age, it was decidedly smaller in the former than in the latter affection. After the age of 50, it was as soft in typhus as in typhoid fever; before that age it was frequently softened."

"*Gall Bladder.* There was ulceration of the lining membrane of the gall bladder in one of fourteen cases of typhoid fever; in none of thirty-one cases of typhus fever. In the latter disease the bile was much thicker, and of a darker green color than in the former."

"*Liver, Pacreas, Kidneys.* These organs were more flabby in the cases of typhus than in those of typhoid fever."

"*Urinary Bladder.* This viscus was ulcerated in one of the cases of typhoid fever—in none of the cases of typhus fever."

"*Pericardium.* This cavity contained a small amount of yellowish transparent serosity in all the cases of typhoid fever examined. The contained serosity was red, from transudation of a solution of hæmatosin, in five of thirty-one cases of typhus fever, in which the pericardium was examined before the termination of the fever.*

"*Heart.* The muscular tissue of this organ was much more frequently and decidedly flabby, and its lining membrane was much more frequently and deeply stained of a dark red color, in the cases of typhus fever, than in those of typhoid fever."

"*Lungs.* Granular and non-granular lobular consolidation were very frequent in the subjects dead from typhoid fever—rare in those dead from typhus fever. The reverse was the fact with reference to congestion of the most depending part of the lung."

"*Pleura.* Recent lymph or turbid serosity was found in six of fifteen cases of typhoid fever—i. e., between half and one-third, or in the proportion of forty per cent. The same lesions not much less in amount were found in two only of thirty-six cases of typhus fever—i. e. one-sixteenth, or in the proportion of 55 per cent."

The foregoing is copied from the Monthly Journal of Medical Science for April, 1850.

Dr. J. adds, after the foregoing parallel of pathological appearances, as follows: "The particulars here briefly recapitulated, and still more those fully detailed in the foregoing papers, appear to me to prove indisputably that the symptoms, course, duration, anatomico-pathological lesions, and the tendency to cadaveric changes, are different in typhoid fever from what they are in typhus fever."

---

* There is evidently a mistake in the last sentence.

